# Children with autism spectrum disorder in high technology medicine environments; a qualitative systematic review of parental perspectives

**DOI:** 10.1186/s13643-023-02440-w

**Published:** 2024-01-18

**Authors:** Emelie Pettersson, Berit Møller Christensen, Ingalill Gimbler Berglund, Elisabeth Nylander, Karina Huus

**Affiliations:** 1 CHILD Research Group, Nursing Department, School of Health and Welfare, Jönköping University, P.O. Box 1026, 551 11 Jönköping, Sweden; 2 CHILD Research Group, Department of Natural Science and Biomedicine, School of Health and Welfare, Jönköping University, Jönköping, Sweden; 3 Jönköping University Library, Jönköping University, Jönköping, Sweden

**Keywords:** Autism spectrum disorders, Children, Experiences, High technology environments, Parents, Procedures

## Abstract

**Background:**

Children with autism spectrum disorders are frequent visitors to high technology environments, and their needs may differ from those of their typically developed peers. Procedures in high technology environments can constitute a challenge for these children and their parents since the environment presents many challenges relevant to the child’s impairments. This systematic review aimed to explore the experiences of children with autism spectrum disorders and their parents during procedures in a high technology environment.

**Methods:**

The following sources were searched for this systematic review: Cochrane CENTRAL Trials, CINAHL, Dentistry and Oral Sciences Source, MEDLINE, PsycINFO, Scopus, and Web of Science Core Collection. The search terms included variants of the following concepts: (1) children with autism spectrum disorder and/or their parents and (2) anesthesia or radiographic departments. Publications were not limited by date or study design.

**Result:**

Out of 13,389 bibliographic records, nine studies were eligible for synthesis. After another search in October 2022, one additional study was eligible for synthesis.None of the studies reported children’s experiences, and all ten reported their parents’ experiences. Only one study was conducted in a radiographic context. Parents’ experiences were both positive and negative and were categorized into two main categories: (1) challenges in a new environment and (2) health care professionals’ approaches.

**Conclusion:**

Studies describing children’s experiences with procedures in high technology environments are lacking. The parents described a need for health care professionals to work in structured ways with their child and to be able to make suitable adaptations.

**Systematic review registration:**

This systematic review was registered in advance on the Open Science Framework, https://doi.org/10.17605/OSF.IO/5TXWJ.

**Supplementary Information:**

The online version contains supplementary material available at 10.1186/s13643-023-02440-w.

## Background

According to the Convention on the Rights of the Child, children and parents have the right to enjoy the highest standard of attainable health care, obtained from information about and participation in health care [1]. Autism spectrum disorder (ASD) is a developmental disability characterized by deficits in social communication, restricted interest, and repetitive behaviors [2]. The prevalence of autism globally is 1% in the population, but rates differ across studies and regions [3], and the symptoms of ASD vary widely and can therefore pose unique challenges to healthcare professionals. The deficits in communication ability due to ASD encompass both verbal and nonverbal communication. According to Kopecky and Broder-Fingert [4], only 23% of children with ASD express their needs verbally. Although the manifestations of ASD impairments vary widely and most children with ASD have challenges with communication, and interactions [5], they can also react differently to sensory stimuli [6]. The most commonly reported difficulty with sensory processing is related to loud noises, followed by tactile stimuli [4]. These impairments can constitute a challenge for health care professionals when leading a child with ASD through a procedure in a sensory-rich environment and interacting with new faces. The combination of all of these factors in high technology environments can cause stress in children with ASD resulting in challenging behaviors [7].

Children with ASD often require health care services such as surgery and radiographic procedures [8], and their needs differ from those of their typically developing peers [9]. One explanation for this might be that children with ASD often have comorbid diagnoses, such as developmental, psychiatric, and/or medical diagnosis, which can lead to more frequent health care contact [8]. Children with ASD often depend on familiar environments and routines. They are also at high risk of developing problem behaviors such as tantrums, rule-breaking, compliance, and anxiety [10].

The high technology environments referenced in this study include those encountered in anesthesia and radiographic departments. These environments are often sensory rich, and the sensory inputs in these environments, such as bright lights, beeping machines, and tactile stimuli differ from those in familiar environments [11]. High technology environments are characterized by short encounters with the patients and demands on health care professionals to rapidly build relationship. Nevertheless, both environments encounter patients in acute need of a procedure, but most of anesthesia and radiology patients have a planned visit, compare with an emergency department. When health care professionals know a child with ASD is coming for a procedure, it is possible to facilitate the procedure for the child, by identifying the individual’s needs in advance.

Understanding the needs of each child with ASD is one way to facilitate the care of these children and their parents. Family-centered care is an approach in which the provided care is based on teamwork, through partnerships between the patients and families and health care professionals. Family-centered care approach encourages family participation by setting goals for care together with health care professionals, which requires open communication among team members [12]. Parenting a child with autism can be challenging, and parents play a key role in supporting children presenting for procedures in a high technology environment. Parents of children with ASD often experience higher levels of stress than parents of children with other disabilities and those of children developing in a typical manner [13]. The characteristics of ASD can also have a negative effect on a family’s quality of life, resulting in a lower state of well-being, more symptoms of depression and higher states of anxiety [14].

According to Bjorkman et al. [15], no radiology departments in Sweden have guidelines for caring for children with ASD, and only a limited number of anesthesia clinics have such guidelines [16]. However, multiple studies have described ways to facilitate the care of these children, and several interventions have been developed for this purpose [17]. The implementation of such interventions is complex, and their successful implementation is essential to facilitate patient care. Successfully implemented interventions often involve multiple components, such as guidelines and education. In addition, co-morbidities can also influence the use of the intervention [18]. Despite the rich body of knowledge regarding facilitation of procedures in different environments for children with ASD, evidence about how children and their parents experiences these procedures is quite sparse.

Therefore, this systematic review aimed to explore the experiences children with ASD, and their parents during procedures in a high technology environment.

## Methods

This systematic review followed a structured process as outlined in the PRISMA reporting guidelines [19]. Systematic reviews can inform practitioners and identify research gaps [20].

### Eligibility criteria

The search structure was informed by the Population, Phenomena of Interest, Context (PICo) framework [21], which has been elucidated below.

- Population: Children 0–18 years old with autism and their parents or primary caregivers.
- Phenomenon of interest: Patient and family experiences.
- Context: Procedures at anesthesia and radiology departments.

The resulting concepts and key terms that informed the search are listed in Table 1.

**Table 1:** Concepts and key terms for search blocks

**Concept blocks**	Children and their primary caregivers’ experiences	Autism (similar diagnoses)	Anesthesia and radiographic departments
Key terms	Children Adolescents Preadolescents Teens Young adults Youths Caregiver Carer Guardian Father Mother Parent Step-father Step-mother Step-parent Family Step-family Kinship network Relative Professional-family relations/relationships	Autism Autistic spectrum disorder ASD Asperger syndrome/disease Asperger’s Disintegrative disorder Pervasive developmental disorder Pervasive child developmental disorder Heller’s syndrome Kanner syndrome Kanner’s syndrome	Anesthesia Anesthesiology Anesthetics Radiograph Radiographic Radiography Radiology X-ray Peri-radiographic Ambulatory surgery Day procedure Day care procedure Medical encounter Medical procedure Perioperative Preoperative Postoperative Post-anesthesia Post-surgical Surgery

The procedure, as defined in this study, begins when a decision is made about the child’s need for anesthesia or radiographic procedures.

No study design limitations were imposed to allow exploration of experiences that could be described in different ways. Furthermore, no limitations regarding the date or language were imposed.

### Information sources

The following databases and/platforms were searched at the beginning of March 2021: CINAHL with Full Text (EBSCOHost), Dentistry and Oral Sciences Source (EBSCOHost), MEDLINE (EBSCOHost), PsycInfo (ProQuest), Scopus (Elsevier), and Web of Science Core Collection (SCI-EXPANDED, SSCI, A&HCI, CPCI-S, CPCI-SSH, and ESCI). In addition, the CENTRAL trial registry of the Cochrane Collaboration (Wiley) was searched for ongoing and recently completed trials. Further information on the coverage dates is provided in Appendix 1. Additional articles were identified by screening the bibliographies and citing the references of the final studies that met the inclusion criteria. The Web of Science Core Collection was used to identify the citing articles.

### Search strategy

The search strategies were developed by a research librarian (EN) and peer reviewed by another information specialist who was not otherwise associated with the research project.Peer review involved proofreading the syntax, spelling, and overall structure but did not use the PRESS checklist [22]. Four studies, which meet the inclusion criteria for the review, were used to identify candidate search terms by looking at the words in the titles, abstract, and subject indices of the studies. A draft search strategy for MEDLINE was developed using those terms and additional search terms were identified from the results of this strategy with input from the project team. The search strategy was validated by testing whether search terms from the four studies could be used to identify candidate search terms. After the MEDLINE strategy was finalized, it was adapted to the syntax and subject headings of the other databases/platforms. See Appendix 1 and 2 for the documentation of all search strategies.

### Selection process

**Fig. 1:**
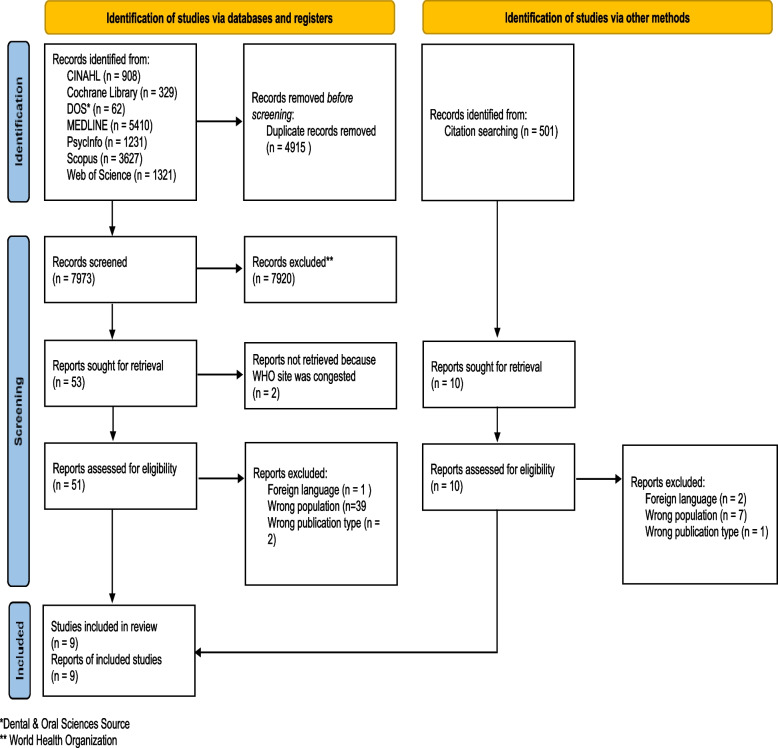
Flow diagram of the screening process renewed search. *Adapted from*: Page MJ, McKenzie JE, Bossuyt PM, Boutron I, Hoffmann TC, Mulrow CD, et al. The PRISMA 2020 statement: an updated guideline for reporting systematic reviews. BMJ 2021;372:n71. https://doi.org/10.1136/bmj.n71. For more information, visit: http://www.prisma-statement.org/

**Fig. 2:**
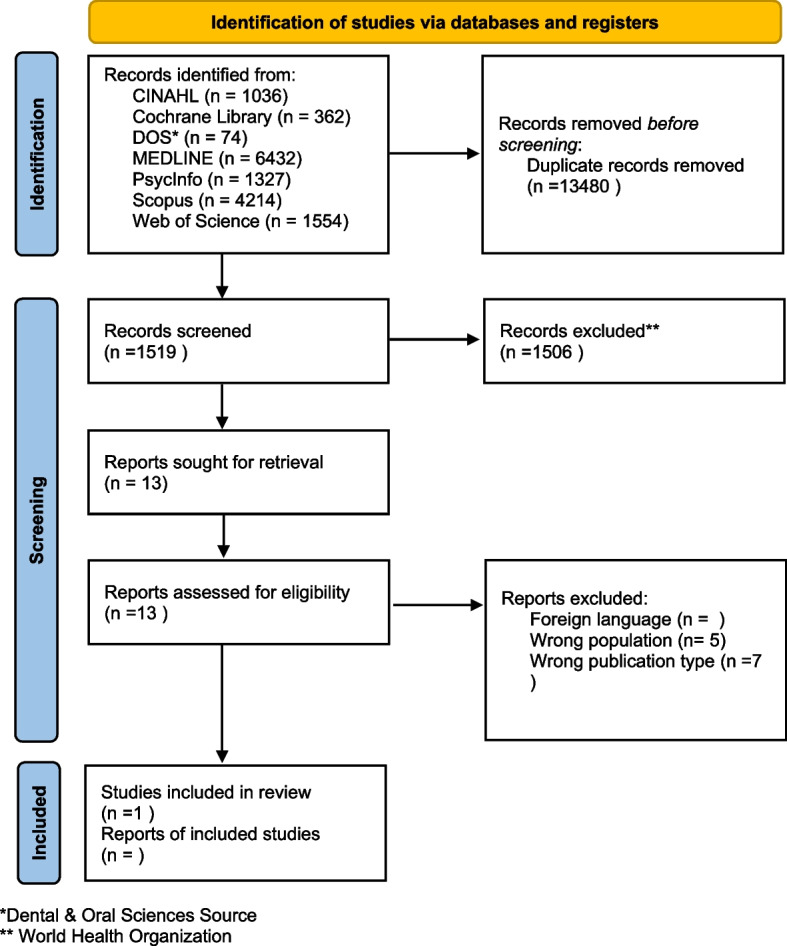
Flow diagram of the screening process first round. Page MJ, McKenzie JE, Bossuyt PM, Boutron I, Hoffmann TC, Mulrow CD, et al. The PRISMA 2020 statement: an updated guideline for reporting systematic reviews. BMJ 2021;372:n71. https://doi.org/10.1136/bmj.n71. For more information, visit: http://www.prisma-statement.org/

Records identified during the search phase were exported to reference management software (EndNote) to enable the identification and removal of duplicates [23] (Fig. 1). Prior to the formal screening process, a calibration exercise was conducted to pilot and refine the screening questions. Records were then screened using Rayyan, a web-based application for systematic reviews [24], based on the previously described inclusion/exclusion criteria. The researchers (EP, BMC, IGB, KH) independently performed the screening process in teams of two at the title/abstract level as well as full-text assessment of included records. Any disagreements during the screening process were resolved through discussion and consensus between the reviewers. An updated search was performed in October 2022 which resulted in 14,999 records, of which 1519 records were screened using Rayyan after removing duplicates (Fig. 2). See Appendix 2 for the documentation of search strategies. The researchers (EP, BMC, IGB, KH) independently performed the screening process in teams of two at the title/abstract level along with the full-text assessment of the included records. The updated search yielded one record that was eligible for synthesis.

The Critical Appraisal Skills Program (CASP) checklist for qualitative studies was used to reflect the quality of the included records (Table 2) [25]. The CASP checklist consists of ten questions divided into three sections: the first section assesses whether the results are valid, the second section characterizes the results of the study, and the third section outlines the potential contribution of the results. The first nine questions can be objectively answered as yes, no, or cannot tell, whereas the last question evaluating the value of the results is more reflective. All records had appropriate methodology and valid results, they had clear statements of the findings, and nine out of ten addressed ethical issues, although they sometimes lacked critical descriptions regarding the researcher’s relationship with the participants and potential bias. After discussions in the research group, all records were assessed to have sufficient quality for inclusion in the review.

**Table 2:** Overview CASP assessment

Study	CASP Criterion 1	CASP Criterion 2	CASP Criterion 3	CASP Criterion 4	CASP Criterion 5	CASP Criterion 6	CASP Criterion 7	CASP Criterion 8	CASP Criterion 9	CASP Criterion 10
	Clear statement of aim	Quality methodology report	Appropriate research design	Sampling	Data collection	Relationship	Ethical issues	Data analysis	Clear statement	Value
Benich et al. (2018) [26]	Yes	Yes	Yes	Yes	Yes	Yes	Yes	Yes	Yes	Yes
Clark et al. (2019) [27]	Yes	Yes	Yes	Yes	Yes	No	No	No	Yes	Yes
Fahy et al. (2020) [28]	Yes	Yes	Yes	Yes	Yes	No	Yes	Unclear	Yes	Yes
Lindberg et al. (2012) [29]	Yes	Yes	Yes	Yes	Yes	No	Yes	Yes	Yes	Yes
Snow et al. (2021) [30]	Yes	Yes	Yes	Yes	Yes	No	Yes	Yes	Yes	Yes
Swartz et al. (2017) [31]	Yes	Yes	Yes	Yes	Yes	No	Yes	No	No	Yes
Taghizadeh et al. (2019) [32]	Yes	Yes	Yes	Yes	Yes	No	Yes	Yes	Yes	Yes
Thompson et al. (2014) [33]	Yes	Yes	Yes	Yes	Yes	Yes	Yes	Yes	Yes	Yes
Tziraki et al. (2021) [34]	Yes	Yes	Yes	Yes	Yes	Unclear	Yes	No	Yes	Yes
Whippey et al. (2019) [35]	Yes	Yes	Yes	Yes	Yes	Unclear	Yes	Unclear	Yes	Yes

### Synthesis of the results

All included records, see Table 3, had a qualitative design and were therefore analyzed using inductive content analysis as explained by Lindgren and Lundman [36]. The use of content analysis in systematic reviews has certain limitations, one of which relates to the generation of data from different epistemological frameworks. A key difference between using content analysis for primary data and a systematic review is that the data in a systematic review are already analyzed, limiting the abstraction level [37], and therefore, the categories in this review contain a low degree of interpretation and abstraction. The study by Lindgren and Lundman [36] was used to guide abstraction and interpretation during the analysis. In this study, the first author (EP) read all included records several times to obtain an overall understanding of the content. After reading all the records, each record was decontextualized by breaking down the results into meaning units according to the aim. The units were then condensed and copied into an Excel spreadsheet by the first author (EP), and recontextualization was started by finding new patterns. All units from the Excel spreadsheet were independently subcategorized by each author (EP, BMC, IGB, KH). After discussing the units and subcategories, new sub-categories emerged, and finally, categories were discussed until a consensus was reached. Content analysis is a nonlinear process in which the authors sometimes return the records during discussions [36].

**Table 3:** Summary of study characteristics and findings

First author, year	Title	Participants/respondents (*n*)	Age children (years)	Country	Design	Data collection	Outcomes
Benich (2018) [26]	Parental Perception of the Perioperative Experience for Children with Autism	12, 10 mothers, 1 father, 1 grandmother	3–16	USA	Qualitative design	Interviews	The study indicates that children with ASD pose a unique challenge to HCPs, and that HPCs need to develop skills for assessing and managing care
Clark (2019) [27]	Improving Communication Between Health Care Providers, Families, and Children with Autism Spectrum Disorder: The Linked Program	31 children	20 months–18 years	USA	Evidence-based practice	Telephone calls with parents	The program enabled a better communication between families and caregivers, and provided awareness to the caregivers of different challenges in the perioperative setting for children and families
Fahy (2020) [28]	Improving peri-operative psychosocial interventions for children with autism spectrum disorder undergoing ENT procedures	25 HCPs, 9 parents	10.33 years + /- 2.199	Ireland	Quantitative and qualitative design	Interviews	The care of each child requires an individualized, parent-led plan
Lindberg (2012) [29]	The experiences of parents of children with severe autism in connection with their children’s anesthetics, in the presence and absence of the perioperative dialogue: a hermeneutic study	12 parents	5–16	Sweden	Qualitative design	Interview	The study indicates continuity in anesthesia care makes a difference. Inviting partners and children in the dialogue makes a difference
Snow (2021) [30]	A balancing act: An interpretive description of healthcare providers and families perspective on the surgical experiences of children with autism spectrum disorder	8 parents 15 HCPs	3–18	Canada	Qualitative design	Interview	The study highlighted a number of factors that create challenges and have a potential to improve the parents and patients experiences, such as importance of collaborative relations between HCP and families, and a need for flexible policies
Swartz (2017) [31]	Benefits of an individualized perioperative plan for children with autism spectrum disorder	124 parents	9.7 +/- 4.0	Canada	Quantitative and Qualitative design	Post-operative contact	The study suggests that implementation of an individualized ASD perioperative management program based on the parents’ input is helpful
Taghizadeh (2019) [32]	The experiences of children with autism spectrum disorder, their caregivers and health care providers during day procedure: A mixed methods study	14 HCP and 15 parents		Australia	Qualitative and quantitative design	Interviews	The study indicates that optimizing care for children with ASD may include changed workflow, staff training and use of aids
Thompson (2014) [33]	Improving Management of Patients with Autism Spectrum Disorder Having Scheduled Surgery: Optimizing Practice	43 caregivers	3–16	USA	Qualitative design	Interview	The study highlights the importance of knowing details about the child behavior and needs prior to the initial contact
Tziraki (2021) [34]	A Neuroimaging Preparation Protocol Tailored for Autism	31 children, 25 caregivers	4, 5–10	Greece	Quantitative and Qualitative design	Open-ended questions	A pediatric patient preparation protocol that included dummy headphones, earplugs, and a social story, successfully facilitated awake MRI imaging with children with ASD
Whippey (2019) [35]	Enhanced perioperative management of children with autism: a pilot study	18 children	3–17	Canada	Qualitative and Quantitative design	Questionnaire, partly with open-ended question	This pilot study highlighted a multidisciplinary perioperative care pathway that improves the perioperative experience for severely autistic children and their families

## Results

### Study selection

A search of the databases/platforms retrieved 12,888 records (Fig. 1). After removing duplicates, 7973 records were screened, of which 53 were full-text documents. Two clinical trial reports were not retrieved because the WHO website had technical issues and the reports could not be retrieved at the time. We then searched for documents that cited any of the initially included studies and the references of the initially included studies, resulting in additional 501 records for screening. However, this did not result in the inclusion of further studies. Nine studies were eligible for analysis based on the review criteria. None of the studies reported the children’s experiences. All nine included studies described the parents’ experiences to different degrees. In cases where the studies included experiences from health care professionals, only the parents’ experiences were coded.

Of the 1519 records found in the updated search, the full texts of nine were screened. One of the screened records was eligible for synthesis [30].

Two other studies, one by Davignon and Friedlaender [38] and the other by Bevan et al. [39] seemed to be eligible for analysis according to the inclusion criteria; however, in the study by Davignon and Friedlander [38], the settings did not match the inclusion criteria for this study. In contrast, Bevan et al. [39] did not explore parents’ experiences of a surgical procedure; rather, the interviews with the parents were used to develop the cases presented in the study [39].

All the included studies were assessed to confirm sufficient quality for inclusion in the review. All of the studies described a clear aim, used appropriate methods, and were designed accordingly. For almost all of the studies, there were questions that could not be accessed in the CASP checklist, due to the lack of descriptions, such as the relationship between the researcher and participants. Furthermore, the researchers’ roles and the risk of bias were not clearly described in the studies.

The parents’ experiences of procedures for their child with autism in a high technology environment could be classified into two main categories: (1) challenges in a new environment, which included four subcategories and (2) health care professionals’ approaches, which included three subcategories (Table 4).

**Table 4:** Main categories and subcategories

Negative consequences of waiting time	Challenges in a new environment
Sensory-rich environment affects the child’s behavior	
Familiar support can help the child to adjust	
Benefits of having a plan	
Communication can be a barrier and a facilitator	Health care professionals’ roles and actions
The attitude makes a difference	
Knowledge has an impact	

### Challenges in a new environment

The category challenges in a new environment included the following subcategories: *negative consequences of waiting times*, *sensory-rich environment affects the child’s behavior*, *familiar support can help the child to adjust,* and *benefits of having a plan* (see Table 4). Nine of the ten studies were included in this category [26–33, 35].

### Negative consequences of waiting times

Waiting times included two aspects, waiting time before receiving a date for the procedure and waiting time before the procedure(34). Parents experienced waiting time as a challenge for children with ASD [26, 28, 32, 35], especially in new environments [26]. Waiting times in the environment led to more exposure to the sensory rich environment and could, in some situations, be directly connected and trigger negative behaviors in the child [26]. In addition, waiting time led to the child becoming hungry and thirsty, which increased the child’s anxiety [32].

### Sensory-rich environment affects the child’s behavior

Children with ASD can be hypersensitive to sensory stimuli, and parents have described how different sensory inputs can negatively affect children with ASD [26, 27, 32]. One of the sensory inputs that was challenging for children with ASD was loud noises, such as crying children, which could trigger different negative behaviors in the children [26, 32]. Commotion near the child could be another stimulus that increases anxiety in the child [26]. For children who have difficulty processing sensory stimuli, the lights in the room could also constitute a problem [27]. Because of these differences in sensory processing, parents also described their child’s different experiences of pain, and the challenges faced by them in expressing their pain [26]. Based on their experiences, parents identified areas for improvement, such as quieter environments and separate rooms for children and their families [28, 32].

### Familiar support can help the child to adjust

Parents described different ways of supporting children with ASD when coming for a procedure in a high technology environment. They stressed the importance of health care professionals reminding and encouraging parents to bring familiar toys from their home. A toy or something else with which the child feels familiar is one way to bring comfort during the procedure [26, 33]. Another support for the child could be the use of familiar tablets or other devices before, during, and after the entire procedure [32, 35]. Children with ASD often have special interests that can also be used as a distraction during the procedure [32]. Parents also described different sensory items [30], child life specialists or play therapists during the procedure as having a positive impact [32, 35].

Parental presence was also mentioned as a support for the child in all steps of the procedure [26, 35], and in some situations, having both parents present during the procedure was beneficial [32]. Parental presence also included parental involvement, wherein parents actively collaborated with health care professionals during the procedure to make things go smoothly [30]. Parent’s unique knowledge about their child, including the needs and measures to support the child [30] can be useful knowledge for health care professionals aiming to support the child during the procedure [26].

Parents described different areas of improvement during the procedure. The first is the use of social storytelling [28, 32]. Social story telling can both reduce anxiety and be a way to individualize care, which can lead to better cooperation. However, it is important to consider that children with severe impairments may not be able to comprehend social stories [32]. Another potential area for improvement, as identified by parents, was for health care professionals to use visual aids to communicate with children [28]. Although the parents identified several adjustments for their child during the procedure, they still wanted their child to be seen and heard [32].

One way to familiarize children with the environment is to limit the number of health care professionals involved with the procedure [26, 29]. Meeting a person the child has met before can have a calming effect. Parents described the child’s willingness to cooperate increased when seeing a familiar face, which also made parents feel more relaxed [29]. Parents also described that a situation with too many health care professionals could lead to increased anxiety in the child and necessitate the use of physical restraint [29].

### Benefits of having a plan

Standard hospital routines for these procedures were not suitable for children with ASD, and parents felt that their child required different routines. One challenge for the child and parents was all the transitions that took place during procedures [28]. Situations could get out of control and parents blamed themselves for not intervening [29].

Many parents described the importance of planning for the day of the procedure [26, 27, 31, 33], described a sense of gratitude when receiving the opportunity to participate in the planning of the child’s procedure [31], and appreciated when their child’s special needs were identified prior to the health care professionals’ first encounter with the child [31, 33]. Parents highlighted the need for health care professionals to follow the plan if a specific plan was made for the child.

Some parents preferred that the child be scheduled for procedures in the morning. This minimized the stress of anticipation and had less effect on eating schedules. Scheduling in the morning also allowed the child to sleep through the preoperative process [26].

### Health care professionals’ roles and actions

The category *health care professionals’ roles and actions* included three subcategories: *communication can be a barrier and a facilitator*, *the attitude makes a difference,* and *knowledge has an impact *(see Table 4). All ten included studies described parents’ experiences in relation to health care professionals [26–35].

### Communication can be a barrier and a facilitator

Parents described communication as an opportunity for health care professionals and children to work together to set rules for the upcoming procedure. Communication between parents and health care professionals could also be an opportunity for parents to share information about their children with health care professionals [29]. Parents described that they felt listened to in communication with health care professionals [34]. Health care professionals gave parents the opportunity to share information about the child’s special needs and listened to what was important for the child [29, 30]. In contrast, some parents also described the communication with health care professionals as talking to deaf ears, implying that the health care professionals did not listen, which forced the parents to repeat all the information provided about the child’s needs. Moreover, in some cases, information provided by the parents to the health care professionals beforehand did not reach the professionals encountering the child on the day of the procedure [29]. The dialogue between parents and health care professionals often remained incomplete or interrupted by telephone calls or changes in the shifts of the health care professionals [29]. Parents also described a lack of necessary information. They wanted more information both about the specific procedure and recovery as well as information about any special considerations in recovery related to the child’s ASD [26].

Parents described communication between the child and health care professionals as a challenge because of the child’s impairment, limited verbal abilities, and individual needs and abilities for communication [26, 28, 29, 32]. Information could be a source of frustration for the child because of the child’s impairments, which imposed limitations in understanding and participating in the communication [29]. Parents reported a connection between too much information beforehand and increased levels of the childs’ anxiety [32]. At the same time, they also experienced a lack of information about the hospital journey of the child [28, 30]. Lack of time was another challenge and became a barrier when encountering a child with ASD. Communication between health care professionals and the child was too sporadic during the day of the procedure, and communication was often interrupted by other things requiring the health care professionals’ attention [29].

Interactions between the child and health care professionals were described as relaxing, and parents felt they could step aside and let the health care professionals lead the child through different situations [29]. The parents appreciated health care professionals efforts to successfully ensure good communication with the child [32], take time to listen, and adapt their communication to the child’s level of understanding [29]. The parents also appreciated when health care professionals were patient with the child and ensured that the child was slowly introduced to situations that could trigger anxiety [26, 35].

Parents described positive impressions for premedication. Unfortunately, the premedication situation often lacks communication regarding the child’s preferences. The lack of communication in these situations made the situation messy and often necessitated physical restraint. As a consequence, the entire experience turned out to be negative one [32].

### The attitude makes a difference

Parents described health care professionals’ behaviors as creative and flexible. Flexible behaviors include not taking observations, such as temperature and blood pressure measurements, if this would make the child anxious [32]. Another example of a flexible approach was not exchanging the child’s own clothes for a hospital gown prior to the procedure. Parents also described flexibility such as letting the child bring a personal toy into the operation area. Creative behaviors were described in relation to premedication and included new ways to administer the premedication to the child and await effect [32].

Nonflexible approaches from health care professionals could have negative consequences for children and procedures. A nonflexible approach could cause behavioral problems in the children. These nonflexible approaches are exemplified by rigid adherence of health care professionals to the protocol [32] without any consideration for the consequences. A lack of patience from a health care professional could be another source of problem for children [28].

The parents experienced that health care professionals made the child feel special, which was a positive experience, and they also felt supported by health care professionals as parents [34]. Parents also described that the health care professionals showed empathy and took good care of their child [27, 33]. In challenging situations, showing empathy could be an opportunity to build relationships between the health care professionals, the children, and their parents [30].

### Knowledge has an impact

Some parents described a lack of knowledge among health care professionals, which manifested as situations wherein health care professionals did not have the tools to support the child during the procedure [28, 29].

The parents appreciated when the health care professionals had knowledge about the specific child and its needs [30] and expressed gratitude for care that was individualized and adapted to those needs [29, 31, 33–35]. Some parents were surprised by individual care [33].

## Discussion

In this systematic review, parents’ experiences were categorized into two main categories: challenges in the new environment and health care professionals’ approaches. The review revealed both positive and negative parent experiences related to the environment and interactions with health care professionals. Both the clinical environment and its health care professionals influence how parents experiences these procedures. However, even though these children are frequent visitors to high technology environments, we did not find any studies describing their experiences in these environments in their own words. This lack of such studies means that the present review only summarizes the experiences of parents of children with ASD.

Many factors affect the experiences of visiting a high technology environment [39]. A common issue identified by this systematic review is the need to make adjustments that focus on the individual needs of a child before entering the high technology environment. Family-centered care is one approach that can be used by health care professionals before, during and after the procedure. This approach enables parents to be an active part of the intra-procedure process and function together with health care professionals as a team [12]. This review revealed that optimal care is provided through the cooperation of the children, their families, and health care professionals. Creating an individual pathway for each child creates demands for both health care professionals and organizations. Health care professionals require knowledge about both ASD in general and the individual child before the encounter to make appropriate adjustments. In the family-centered care approach, both children and their parents should be engaged before the procedure to identify the individual needs of the child [40]. Davignon and Friedlaender [38] identified health care professionals’ lack of knowledge as a barrier for interactions between them and the child. Nicholas, Muskat [41] also described the importance of health care professionals’ knowledge regarding both ASD and also the individual child before the health care professionals encounter the child and the family. In this regard, another important consideration is the possibility of health care systems to identify children with ASD ahead of time in order to enable planning before an encounter [10, 38]. Working together with families can facilitate important aspects of care, such as continuity of care, partnership in decision-making, and the ability to adjust to the child’s specific needs [17, 41].

Despite the multiple benefits of making specific adjustments for each child, there also seem to be some general recommendations that can facilitate care for this population. One such facilitator is minimizing the time that the child is in the environment. There are several aspects of waiting that can affect the child, such as to many sensory inputs, feelings of hunger and thirst, and meeting many new faces. The overall effect of these factors could be an increase in the child’s anxiety level. Wilson and Peterson [11] also described the challenging environment in health care settings that can lead to difficult child behavior, which may affect the care the child receives. Facilitators, as described by Wilson and Peterson [11], Koski, Gabriels [17], also enable children to communicate and health care professionals to listen. Limiting the child’s possibility to communicate often leads to frustration and increased anxiety. The parents in this review described the need for health care professionals to use aids to communicate with their children. Møller christensen and Nilsson [42] highlighted the need for health care professionals to be flexible in their communication with these children and their parents during health care procedures and found that illustrations can be a tool to facilitate communication adapted to the individual child. According to the Convention on the Rights of the Child [1], children have the right to express their views, and for some children, this requires communication using various aids. According to the same convention, children also have the right to access the highest attainable standard of health care. Encountering children with ASD and their families does set high demands on health care professionals. A flexible health care professional who can both follow and lead the child through the procedure can serve as a facilitator, for both the child and its parents. Future studies should consider evaluating children’s experiences during a procedure in high technology environments. Furthermore, this study revealed the major impact of health care professionals; thus, studies exploring health care professionals’ experiences with these procedures could be of great interest.

### Limitations

Despite the broad inclusion criteria of this systematic review, only ten studies were included in the analysis. None of the studies described the children’s experiences. No quantitative studies measuring the experiences were identified, and the lack of quantitative data precluded statistical calculations. This review was limited to the anesthesia and radiology departments. One study was conducted in the radiographic context and nine studies were conducted in the anesthesia context. Although the literature search was performed without any limit regarding the year of publication, all included studies were published in the past 10 years. This review explores the specific environments anesthesia and/or radiology departments. However, several other health care settings can also constitute a challenge for children with ASD and their families.

## Conclusions

Health care professionals and the clinical environment are factors that affect children with ASD and their parents’ experiences of a procedure. Health care professionals, based on their knowledge, attitudes, and communication skills, can constitute both a facilitator and a barrier to a child’s way through a procedure. Parents described the need for making suitable adaptions for the child before and during the procedure in these challenging environments.

### Supplementary Information

**Additional file 1. **

**Additional file 2. **

## Data Availability

The datasets used and/or analyzed during the current study are available from the corresponding author on reasonable request.
