# Supplementary material for: Children with autism spectrum disorder in high technology medicine environments; a qualitative systematic review of parental perspectives
Source: Syst Rev. 2024 Jan 18;13:34. doi: 10.1186/s13643-023-02440-w (PMC10795331; doi:10.1186/s13643-023-02440-w)
Supplement: Supplementary file 1 — Additional file 1. [file 13643_2023_2440_MOESM1_ESM.docx]

Appendix 1

# Supplemental material – Search strategies

CINAHL with Full Text (EBSCOhost) n=908

Date of final search: March 1, 2021
Years of coverage: 1976 - 2021

| **#** | **Query** | **Results** |
| --- | --- | --- |
| S1 | (MH "Child") OR (MH "Adolescence") OR (MH "Child, Preschool") | 856,846 |
| S2 | child* OR adolescen* OR preadolescen* OR teen* OR “young adult*” OR youth* | 1,255,825 |
| S3 | (MH "Caregiver Attitudes") OR (MH "Caregivers”) OR (MH "Parents+") OR (MH "Family+") OR (MH "Professional-Family Relations") | 271,270 |
| S4 | caregiver* OR “care giver*” OR carer* OR guardian* OR parent* OR father* OR mother* OR step-parent* OR stepparent* OR step-father* OR stepfather* OR step-mother* OR stepmother* OR relative* OR family* OR families* OR step-family* OR stepfamily* OR step-families* OR stepfamilies* OR “kinship network*” OR (professional N3 family N3 relation*) | 788,497 |
| S5 | S1 OR S2 OR S3 OR S4 | 1,734,619 |
| S6 | (MH "Child Development Disorders, Pervasive+") | 27,338 |
| S7 | autism OR autistic OR asd OR asperger* OR disintegrative OR kanner* OR heller* OR “pervasive child developmental” OR “pervasive developmental” | 38,046 |
| S8 | S6 OR S7 | 38,445 |
| S9 | (MH "Anesthesiology Service") OR (MH "Anesthesia+") OR (MH "Anesthesiology") OR (MH "Anesthetics+") | 80,352 |
| S10 | anesthesia OR anesthesiology OR anesthetic* OR anaesthesia OR anaesthesiology OR anaestethic* | 83,630 |
| S11 | (MH "Radiology Service") OR (MH "Radiography+") | 211,681 |
| S12 | peri-radiograph* OR periradiograph* OR radiograph* OR radiology OR x-ray* | 265,158 |
| S13 | (MH "Ambulatory Surgery") | 6,714 |
| S14 | (day N3 procedure*) OR (medical N3 (procedure* OR encounter*)) | 7,342 |
| S15 | (MH "Anesthesia Recovery") OR (MH "Perioperative Care") OR (MH "Postoperative Care") OR (MH "Postoperative Period") OR (MH "Preoperative Care") OR (MH "Preoperative Period") | 62,008 |
| S16 | peri-operative OR perioperative OR pre-operative OR preoperative OR post-anesthesia OR postanesthesia OR post-anaesthesia OR postanaesthesia OR post-operative OR postoperative OR post-surgery OR post-surgical OR surgery OR surgical | 720,390 |
| S17 | S9 OR S10 OR S11 OR S12 OR S13 OR S14 OR S15 OR S16 | 997,919 |
| S18 | S5 AND S8 AND S17  Limiters - Peer Reviewed Search modes - Boolean/Phrase | 908 |

## Cochrane Library (Wiley) n=329

Date of final search: March 2, 2021
Years of coverage: 1996 – 2021

**ID Search Hits**

#1 MeSH descriptor: [Child] explode all trees 57420

#2 MeSH descriptor: [Adolescent] explode all trees 105819

#3 child* OR adolescen* OR preadolescen* OR teen* OR “young adult*” OR youth* 320888

#4 MeSH descriptor: [Caregivers] this term only 2225

#5 MeSH descriptor: [Parents] explode all trees 5230

#6 MeSH descriptor: [Family] explode all trees 9592

#7 MeSH descriptor: [Professional-Family Relations] this term only 216

#8 caregiver* OR “care giver*” OR carer* OR guardian* OR parent* OR father* OR mother* OR step-parent* OR stepparent* OR step-father* OR stepfather* OR step-mother* OR stepmother* OR relative* OR family* OR families* OR step-family* OR stepfamily* OR step-families* OR stepfamilies* OR “kinship network*” OR (professional NEAR/3 family NEAR/3 relation*) 199328

#9 (35-#8) 444930

#10 MeSH descriptor: [Child Development Disorders, Pervasive] explode all trees 1741

#11 autism OR autistic OR asd OR asperger* OR disintegrative OR kanner* OR heller* OR “pervasive child developmental” OR “pervasive developmental” 6589

#12 #10 OR #11 6594

#13 MeSH descriptor: [Anesthesia Department, Hospital] this term only 6

#14 MeSH descriptor: [Anesthesia] explode all trees 19689

#15 MeSH descriptor: [Anesthesiology] this term only 420

#16 MeSH descriptor: [Anesthetics] explode all trees 16257

#17 anesthesia OR anesthesiology OR anesthetic* OR anaesthesia OR anaesthesiology OR anaestethic* 98066

#18 MeSH descriptor: [Radiology Department, Hospital] this term only 26

#19 MeSH descriptor: [Radiography] explode all trees 21045

#20 MeSH descriptor: [Radiology] explode all trees 199

#21 peri-radiograph* OR periradiograph* OR radiograph* OR radiology OR x-ray* 50503

#22 MeSH descriptor: [Day Care, Medical] this term only 254

#23 (day NEAR/3 procedure*) OR (medical NEAR/3 (procedure* OR encounter*)) 3720

#24 MeSH descriptor: [Anesthesia Recovery Period] this term only 2059

#25 MeSH descriptor: [Perioperative Care] this term only 1025

#26 MeSH descriptor: [Perioperative Period] this term only 254

#27 MeSH descriptor: [Preoperative Care] this term only 4309

#28 MeSH descriptor: [Preoperative Period] this term only 316

#29 MeSH descriptor: [Postoperative Care] this term only 4539

#30 MeSH descriptor: [Postoperative Period] this term only 4081

#31 peri-operative OR perioperative OR pre-operative OR preoperative OR post-anesthesia OR postanesthesia OR post-anaesthesia OR postanaesthesia OR post-operative OR postoperative OR post-surgery OR post-surgical OR surgery OR surgical 309116

#32 (36-#31) 380641

#33 #9 AND #12 AND #32 333

**103 Cochrane Reviews** matching "#33 - #9 AND #12 AND #32"

**13 Cochrane Protocols** matching "#33 - #9 AND #12 AND #32"

**213 Trials** matching "#33 - #9 AND #12 AND #32"

## DOS – Dentistry & Oral Sciences Source (EBSCOHost) n=62

Date of final search: March 1, 2021
Years of coverage: 1919 - 2021

| **#** | **Query** | **Results** |
| --- | --- | --- |
| S1 | DE "CHILDREN" OR DE "TEENAGERS" | 4,258 |
| S2 | child* OR adolescen* OR preadolescen* OR teen* OR “young adult*” OR youth* | 38,538 |
| S3 | DE "CAREGIVER attitudes" OR DE "PARENT attitudes" OR DE "ATTITUDES of mothers" OR DE "FATHERS' attitudes" OR DE "CAREGIVERS" OR DE "MALE caregivers" OR DE "WOMEN caregivers" OR DE "FAMILIES" OR DE "PARENTS" OR DE "ADOPTIVE parents" OR DE "BIRTHPARENTS" OR DE "CO-parents" OR DE "DIVORCED parents" OR DE "FATHERS" OR DE "FOSTER parents" OR DE "GRANDPARENTS as parents" OR DE "MOTHERS" OR DE "PARENTS of autistic children" OR DE "PARENTS of children with disabilities" OR DE "PARENTS of people with disabilities" OR DE "SINGLE parents" OR DE "STEPPARENTS" OR DE "TEENAGE parents" OR DE "HEADS of households" OR DE "MEDICAL personnel-caregiver relationships" | 1,313 |
| S4 | caregiver* OR “care giver*” OR carer* OR guardian* OR parent* OR father* OR mother* OR step-parent* OR stepparent* OR step-father* OR stepfather* OR step-mother* OR stepmother* OR relative* OR family* OR families* OR step-family* OR stepfamily* OR step-families* OR stepfamilies* OR “kinship network*” OR (professional N3 family N3 relation*) | 28,374 |
| S5 | S1 OR S2 OR S3 OR S4 | 58,071 |
| S6 | DE "ASPERGER'S syndrome in children" OR DE "AUTISM in children" OR DE "AUTISM spectrum disorders in children" OR DE "AUTISTIC children" OR DE "CHILDHOOD disintegrative disorder" OR DE "CHILDREN with autism spectrum disorders" | 128 |
| S7 | autism OR autistic OR asd OR asperger* OR disintegrative OR kanner* OR heller* OR “pervasive child developmental” OR “pervasive developmental” | 662 |
| S8 | S6 OR S7 | 662 |
| S9 | DE "ANESTHESIA" OR DE "ANESTHESIOLOGY" | 2,214 |
| S10 | anesthesia OR anesthesiology OR anesthetic* OR anaesthesia OR anaesthesiology OR anaestethic* | 12,623 |
| S11 | DE "MEDICAL radiography" OR DE "MEDICAL radiology" OR DE "PEDIATRIC radiography" OR DE "PEDIATRIC radiology" OR DE "RADIOLOGY" OR DE "RADIOGRAPHY" | 5,537 |
| S12 | peri-radiograph* OR periradiograph* OR radiograph* OR radiology OR x-ray* | 51,567 |
| S13 | DE "DAY hospitals" | 1 |
| S14 | (day N3 procedure*) OR (medical N3 (procedure* OR encounter*)) | 17,589 |
| S15 | DE "POSTOPERATIVE care" OR DE "POSTOPERATIVE period" OR DE "PREOPERATIVE care" OR DE "PREOPERATIVE period" | 2,321 |
| S16 | peri-operative OR perioperative OR pre-operative OR preoperative OR post-anesthesia OR postanesthesia OR post-anaesthesia OR postanaesthesia OR post-operative OR postoperative OR post-surgery OR post-surgical OR surgery OR surgical | 137,137 |
| S17 | S9 OR S10 OR S11 OR S12 OR S13 OR S14 OR S15 OR S16 | 171,684 |
| S18 | S5 AND S8 AND S17 Search modes - Boolean/Phrase | 62 |

## MEDLINE (EBSCOhost) n=5,410

Date of final search: March 1, 2021
Years of coverage: 1809 - 2021

| **#** | **Query** | **Results** |
| --- | --- | --- |
| S1 | (MH "Child") OR (MH "Adolescent") OR (MH "Child, Preschool") | 3,076,810 |
| S2 | child* OR adolescen* OR preadolescen* OR teen* OR “young adult*” OR youth* | 4,336,165 |
| S3 | (MH "Caregivers") OR (MH "Parents+") OR (MH "Family+") OR (MH "Professional-Family Relations") | 359,829 |
| S4 | caregiver* OR “care giver*” OR carer* OR guardian* OR parent* OR father* OR mother* OR step-parent* OR stepparent* OR step-father* OR stepfather* OR step-mother* OR stepmother* OR relative* OR family* OR families* OR step-family* OR stepfamily* OR step-families* OR stepfamilies* OR “kinship network*” OR (professional N3 family N3 relation*) | 3,228,355 |
| S5 | S1 OR S2 OR S3 OR S4 | 6,764,945 |
| S6 | (MH "Child Development Disorders, Pervasive+") | 37,164 |
| S7 | autism OR autistic OR asd OR asperger* OR disintegrative OR kanner* OR heller* OR “pervasive child developmental” OR “pervasive developmental” | 89,400 |
| S8 | S6 OR S7 | 89,893 |
| S9 | (MH "Anesthesia Department, Hospital") OR (MH "Anesthesia+") OR (MH "Anesthesiology") OR (MH "Anesthetics+") | 254,677 |
| S10 | anesthesia OR anesthesiology OR anesthetic* OR anaesthesia OR anaesthesiology OR anaestethic* | 538,644 |
| S11 | (MH "Radiology Department, Hospital") OR (MH "Radiography+") OR (MH "Radiology+") | 1,159,432 |
| S12 | peri-radiograph* OR periradiograph* OR radiograph* OR radiology OR x-ray* | 1,655,479 |
| S13 | (MH "Day Care, Medical") | 5,139 |
| S14 | (day N3 procedure*) OR (medical N3 (procedure* OR encounter*)) | 24,993 |
| S15 | (MH "Anesthesia Recovery Period") OR (MH "Perioperative Care") OR (MH "Perioperative Period") OR (MH "Postoperative Care") OR (MH "Postoperative Period") OR (MH "Preoperative Care") OR (MH "Preoperative Period") | 188,187 |
| S16 | peri-operative OR perioperative OR pre-operative OR preoperative OR post-anesthesia OR postanesthesia OR post-anaesthesia OR postanaesthesia OR post-operative OR postoperative OR post-surgery OR post-surgical OR surgery OR surgical | 4,066,714 |
| S17 | S9 OR S10 OR S11 OR S12 OR S13 OR S14 OR S15 OR S16 | 5,715,059 |
| S18 | S5 AND S8 AND S17 Search modes - Boolean/Phrase | 5,410 |

##

## PsycInfo (ProQuest) n=1,231

Date of final search: March 2, 2021
Years of coverage: 1806 - 2021

((MAINSUBJECT.EXACT("Child Attitudes") OR MAINSUBJECT.EXACT("Adolescent Attitudes") OR MAINSUBJECT.EXACT("Caregivers") OR MAINSUBJECT.EXACT.EXPLODE("Parents") OR MAINSUBJECT.EXACT("Family")) OR (noft(child*) OR noft(adolescen*) OR noft(preadolescen*) OR noft(teen*) OR (noft("young adult") OR noft("young adulthood") OR noft("young adults")) OR noft(youth*) OR noft(caregiver*) OR (noft("care giver") OR noft("care givers")) OR noft(carer*) OR noft(guardian*) OR noft(parent*) OR noft(father*) OR noft(mother*) OR noft(step-parent*) OR noft(stepparent*) OR noft(step-father*) OR noft(stepfather*) OR noft(step-mother*) OR noft(stepmother*) OR noft(relative*) OR noft(family*) OR noft(families*) OR noft(step-family*) OR noft(stepfamily*) OR noft(step-families*) OR noft(stepfamilies*) OR (noft("kinship network") OR noft("kinship networks")) OR (noft(professional) N/3 noft(family) N/3 noft(relation*)))) AND (MAINSUBJECT.EXACT.EXPLODE("Autism Spectrum Disorders") OR (noft(autism) OR noft(autistic) OR noft(asd) OR noft(asperger*) OR noft(disintegrative) OR noft(kanner*) OR noft(heller*) OR noft(“pervasive child developmental”) OR noft(“pervasive developmental”))) AND ((MAINSUBJECT.EXACT("Anesthesia (Feeling)") OR MAINSUBJECT.EXACT("Anesthesiology") OR MAINSUBJECT.EXACT.EXPLODE("Anesthetic Drugs")) OR (noft(anesthesia) OR noft(anesthesiology) OR noft(anesthetic*) OR noft(anaesthesia) OR noft(anaesthesiology) OR noft(anaestethic*) OR noft(peri-radiograph*) OR noft(periradiograph*) OR noft(radiograph*) OR noft(radiology) OR noft(x-ray*) OR (noft(day) N/3 noft(procedure*)) OR (noft(medical) N/3 (noft(procedure*) OR noft(encounter*))) OR noft(peri-operative) OR noft(perioperative) OR noft(pre-operative) OR noft(preoperative) OR noft(post-anesthesia) OR noft(postanesthesia) OR noft(post-anaesthesia) OR noft(postanaesthesia) OR noft(post-operative) OR noft(postoperative) OR noft(post-surgery) OR noft(post-surgical) OR noft(surgery) OR noft(surgical)))

Limits applied: Peer-reviewed

##

## Scopus (Elsevier) n=3,627

Date of final search: March 1, 2021
Years of coverage: 1788 - 2021

TITLE-ABS-KEY ( child* OR adolescen* OR preadolescen* OR teen* OR "young adult*" OR youth* OR caregiver* OR "care giver*" OR carer* OR guardian* OR parent* OR father* OR mother* OR step-parent* OR stepparent* OR step-father* OR stepfather* OR step-mother* OR stepmother* OR relative* OR family* OR families* OR step-family* OR stepfamily* OR step-families* OR stepfamilies* OR "kinship network*" OR ( professional W/3 family W/3 relation* ) ) AND TITLE-ABS-KEY ( autism OR autistic OR asd OR asperger* OR disintegrative OR kanner* OR heller* OR "pervasive child developmental" OR "pervasive developmental" ) AND TITLE-ABS-KEY ( anaesthesia OR anaesthesiology OR anaestethic* OR peri-radiograph* OR periradiograph* OR radiograph* OR radiology OR x-ray* OR ( day W/3 procedure* ) OR ( medical W/3 ( procedure* OR encounter* ) ) OR peri-operative OR perioperative OR pre-operative OR preoperative OR post-anesthesia OR postanesthesia OR post-anaesthesia OR postanaesthesia OR post-operative OR postoperative OR post-surgery OR post-surgical OR surgery OR surgical )

## Web of Science Core Collection n=1,321

Date of final search: March 2, 2021
Years of coverage: 1945 - 2021

TOPIC: (child* OR adolescen* OR preadolescen* OR teen* OR “young adult*” OR youth* OR caregiver* OR “care giver*” OR carer* OR guardian* OR parent* OR father* OR mother* OR step-parent* OR stepparent* OR step-father* OR stepfather* OR step-mother* OR stepmother* OR relative* OR family* OR families* OR step-family* OR stepfamily* OR step-families* OR stepfamilies* OR “kinship network*” OR (professional NEAR/3 family NEAR/3 relation*)) AND TOPIC: (autism OR autistic OR asd OR asperger* OR disintegrative OR kanner* OR heller* OR “pervasive child developmental” OR “pervasive developmental”) AND TOPIC: (anesthesia OR anesthesiology OR anesthetic* OR anaesthesia OR anaesthesiology OR anaestethic* OR peri-radiograph* OR periradiograph* OR radiograph* OR radiology OR x-ray* OR (day NEAR/3 procedure*) OR (medical NEAR/3 (procedure* OR encounter*)) OR peri-operative OR perioperative OR pre-operative OR preoperative OR post-anesthesia OR postanesthesia OR post-anaesthesia OR postanaesthesia OR post-operative OR postoperative OR post-surgery OR post-surgical OR surgery OR surgical)

Timespan: All years.
Indexes: SCI-EXPANDED, SSCI, A&HCI, CPCI-S, CPCI-SSH, ESCI.
